# Mid-Season Vaccine Effectiveness Estimates for Influenza: The Department of Defense Global Respiratory Pathogen Surveillance Program, 2024–2025 Season

**Published:** 2025-05-01

**Authors:** Bismark Kwaah, Laurie S. DeMarcus, Jeffrey W. Thervil, Whitney N. Jenkins, William E. Gruner, Tamara R. Hartless, Victor K. Heh, Deanna Muehleman, Anthony C. Fries, Fabrice E. Evengue

**Affiliations:** Defense Centers for Public Health–Dayton, Defense Health Agency, Wright-Patterson Air Force Base, Dayton, OH; JYG Innovations, LLC, Dayton; Innovative Element, LLC, Beavercreek, OH; U.S. Air Force School of Aerospace Medicine Epidemiology Laboratory, Wright-Patterson Air Force Base: Dr. Fries

The Department of Defense Global Respiratory Pathogen Surveillance Program (DoDGRPSP) is an active sentinel respiratory surveillance program that resides at the Defense Centers for Public Health–Dayton (DCPH-D), located at Wright-Patterson Air Force Base. The DoDGRPSP conducts influenza vaccine effectiveness (VE) among Military Health System (MHS) beneficiaries at 118 sites worldwide.

This mid-season analysis includes respiratory specimens from MHS beneficiaries who sought outpatient medical care from November 24, 2024 through March 15, 2025 at a military hospital or clinic and met the influenza-like illness (ILI) case definition. DoDGRPSP methods, including ILI case definition, questionnaire submission, vaccination ascertainment, specimen collection, transportation, testing, and sequencing, have been previously described. ^1^

A test-negative, case-control study design was used to estimate influenza VE against symptomatic laboratory-confirmed influenza. Cases were defined as an ILI patient testing positive for any influenza virus (sub)type with control specimens that had tested negative for influenza. Vaccinated patients were those receiving the 2024-2025 influenza vaccine at least 14 days before symptom onset. Those vaccinated less than 14 days before specimen collection were excluded. VE methodology has previously been described. ^2 , 3^

Specimens were analyzed at Landstuhl Regional Medical Center, Incirlik AB, and DCPH-D by real-time reverse transcriptase-polymerase chain reaction (RT-PCR) and/or viral culture (at DCPH-D only). VE analyses were conducted for influenza A (any subtype), influenza A(H1N1)pdm09, and A(H3N2) for all beneficiaries, adults as well as children. VE estimates were adjusted for confounding factors such as age group, month of illness, and geographic region. Service members, patients less than 6 months old, and individuals with unknown vaccination status were excluded from VE analysis. The analysis included 295 participants who tested positive for influenza and 965 controls who tested negative for influenza.

Adjusted VE estimates among all beneficiaries for influenza A (any subtypes), influenza A(H1N1)pdm09, and influenza A(H3N2) were 25% (95% confidence interval [CI] -1, 44), 58% (95% CI, 31, 74), and 42% (95% CI, 14, 62), respectively. Adjusted VE for children was 27% (95% CI, -5, 50), 69% (95% CI, 43, 83), and 36% (95% CI, -5, 61), while among adults it was 17% (95% CI, -35, 49), 37% (95% CI, -43, 72), and 52% (95% CI, 2, 76) for influenza A (any subtype), influenza A(H1N1)pdm09, and influenza A(H3N2), respectively. VE for influenza B was not calculated due to a small number of cases.

This study reports low to moderate VE, but not all estimates were significant. There was moderate effectiveness against influenza A(H1N1)pdm09 in all beneficiaries and children ages 6 months to 17 years. In adults (ages 18-64 years) and all beneficiaries there was moderate effectiveness against influenza A(H3N2). VE estimates against influenza A (any subtype) for all beneficiaries, children, and adults were non-significant and not effective among children for influenza A(H3N2) and in adults for influenza A(H1N1)pdm09.

**TABLE.** Influenza Vaccine Effectiveness Against Symptomatic Laboratory-Confirmed Ambulatory Influenza, Military Health System Beneficiaries, 2024–2025 Season

Type of Influenza	Population	Vaccinated	Cases		Controls		Crude VE (95% CI)	Adjusted VE ^a^ (95% CI)
			No.	%	No.	%		
A (any subtype)	Adults	Yes	56	61.5	217	64.2	11 (-44, 45)	17 (-35, 49)
		No	35	38.5	121	35.8		
A (any subtype)	Children	Yes	136	66.7	434	69.2	11 (-25, 36)	27 (-5, 50)
		No	68	33.3	193	30.8		
A (any subtype)	All beneficiaries	Yes	192	65.1	651	67.5	10 (-18, 32)	25 (-1, 44)
		No	103	34.9	314	32.5		
A(H1N1)pdm09	Adults	Yes	14	53.8	217	64.2	35 (-45, 71)	37 (-43, 72)
		No	12	46.2	121	35.8		
A(H1N1)pdm09	Children	Yes	21	45.7	434	69.2	63 (32, 80)	69 (43, 83)
		No	25	54.3	193	30.8		
A(H1N1)pdm09	All beneficiaries	Yes	35	48.6	651	67.5	54 (26, 72)	58 (31, 74)
		No	37	51.4	314	32.5		
A(H3N2)	Adults	Yes	18	46.2	217	64.2	52 (7, 75)	52 (2, 76)
		No	21	53.8	121	35.8		
A(H3N2)	Children	Yes	51	55.4	434	69.2	45 (14, 65)	36 (-5, 61)
		No	41	44.6	193	30.8		
A(H3N2)	All beneficiaries	Yes	69	52.7	651	67.5	46 (22, 63)	42 (14, 62)
		No	62	47.3	314	32.5		

Abbreviations: CI, confidence interval; No., number; VE, vaccine effectiveness.

a Adjusted for age group, geographic region and month of illness.
